# Endodontic retreatment of curved canals using Reciproc, Hyflex CM and Prodesign Duo Hybrid Systems

**DOI:** 10.1590/1807-3107bor-2025.vol39.061

**Published:** 2025-06-02

**Authors:** Amanda Garcia Alves MALIZA, Renata Maíra de Souza LEAL, Bruno Cavalini CAVENAGO, Murilo Priori ALCALDE, Rodrigo Ricci VIVAN, Marco Antônio Húngaro DUARTE

**Affiliations:** (a) Universidade de São Paulo – USP, School of Dentistry of Bauru, Department of Operative Dentistry, Endodontics and Dental Materials, Bauru, SP, Brazil; (b) Universidade Federal do Paraná – UFPR, Department of Restorative Dentistry, Curitiba, PR, Brazil.

**Keywords:** Description: Retreatment, Tomography, X-Ray Computed, Confocal Microscopy

## Abstract

The aim of this ex vivo study was to evaluate the efficacy of the Reciproc, Hyflex CM, and ProDesign Duo Hybrid in the retreatment of mesial root canals with Vertucci Type 4 of mandibular molars with curvatures between 10 and 20 degrees. Forty-five mesial roots of mandibular molars were prepared up to size 25.06 Nickel-Titanium (NiTi) file and filled by the single cone technique. The roots were then divided into 3 groups according to retreatment procedures: Reciproc R25 followed by R40; Hyflex 25.04 followed by 40.04; ProDesign Duo Hybrid 25.06 followed by 40.05. The canals were irrigated with 1 mL 1% sodium hypochlorite, and a final rinse was performed with 5 mL 17% EDTA for 3 minutes. Microcomputed tomography scans were performed in each step of the procedure to evaluate root canal transportation at distances of 2, 4, 6, 8 and 10 mm from the apex and volume of endodontic filling remaining at segments of 1–3, 3–5, and 5–7 mm from the apex. The filling material that was not removed during retreatment was evaluated by confocal laser scanning and the percentage after each procedure was expressed in terms of percentage of the initial root-filling material volume. The time taken to remove the material and re-instrumentation was measured in seconds with a digital chronometer. Statistical analysis was performed with t-paired, ANOVA, Kruskal-Wallis and Friedman tests (p < 0.05). In all groups, sections at 4, 6, 8, and 10 mm from the apex were transported to the danger zone, towards the furcation. However, the section at 2 mm from the apex was transported in opposite direction to the furcation, but no statistically significant differences (p > 0.05) in canal transportation and filling material removal were found between the 3 groups. Filling removal and re-instrumentation took significantly (p < 0.05) less time in the Reciproc group. None of the retreatment techniques completely removed the root fillings. The results suggest that no difference exists between the groups in apical transportation of curved canals. The Reciproc system took less time for filling removal and re-instrumentation and the combination of reciprocating and rotary files in the retreatment of curved canals was as efficient as rotary and reciprocating motion.

## Introduction

The primary factor contributing to the failure of endodontic treatment is the persistence of bacteria within the root canal system, leading to intraradicular or extraradicular infections.^1,2^ Retreatment procedures are usually indicated when a canal was left untreated or when an obturated root canal is below the standard requirements. Another retreatment indication is the long-term exposure of the root canal filling material to the oral cavity^3^ due to a secondary infection.^4^ For this purpose, a new access to the canal is required, followed by the removal of the filling material, disinfection of the canal space, and a new root canal filling.^5,6^

The incomplete removal of the filling materials can impact retreatment outcomes since the presence of root canal obturation materials may obstruct the irrigation solutions from coming into contact with persistent microorganisms and by prevent a satisfactory adaptation of the new filling material. Several techniques have been used for removing the root canal filling, including stainless steel hand files^7^ and rotary and reciprocating files, which allow a faster procedure with effective removal of the root canal filling.^8-11^

Reciprocating kinematics emerged as a result of an evolution of the mechanized systems, indicated for root canal instrumentation and removal of filling material in endodontic retreatments, usually performed with a single instrument.^8,10,12^ An adaptive motion system that merges the benefits of both the continuous rotation and reciprocating motion has been implemented^13^. The system allows the instrument to engage in an intermittent motion in the presence of a small load, favoring cutting efficiency and debris removal.^13-15^ Conversely, in the presence of a higher load while cutting dentin, the motion changes to reciprocating rotation, favoring fatigue resistance and minimizing instrument screwing.^13-15^

The conventional NiTi alloy has an austenite structure, but an M-wire NiTi was developed by submitting the material to a thermal treatment, promoting a mixture of nearly equal amounts of R-phase and austenite. This alloy contains substantial amounts of martensite that does not undergo phase transformation, resulting in a metallurgical microstructure alloy of higher strength,^16^ which is the metal alloy used in the Reciproc system (VDW, Munich, Germany). Besides this M-wire technology, another innovative approach is the use of a controlled memory alloy (CM-wire) to produce rotary NiTi instruments with high fracture resistance.^17^

Previous studies have compared the retreatment ability of several rotary, reciprocating, and adaptive motion systems^16-12,18-20^and the literature is still controversial as to the best kinematics for retreatment. So, the aim of this study was to evaluate the influence of rotary, reciprocating, and hybrid motion cinematics of the rotary CM wire files Hyflex CM (Coltene-Whaledent, Allstätten, Switzerland) and ProDesign Duo Hybrid (Easy Equipamentos Odontológicos, Belo Horizonte, Brazil) and reciprocating file Reciproc on the retreatment of curved canals of mandibular molars using micro-CT and confocal laser scanning microscope.

## Methods

### Sample size

The sample calculation was performed using G * Power v. 31 for Mac by selecting fixed effects ANOVA. The data from a previous study (Three-dimensional evaluation of effectiveness of hand and rotary instrumentation for retreatment of canals filled with different materials)^2 2^ was used. The effect size of 0.80 was utilized in the present study. The alpha error was 0.05, and the beta power was 0.90. A total of 12 specimens were required for the group, but fifteen teeth were utilized in case any specimen was lost.

### Teeth selection

After ethics committee approval (protocol 1.334.911), forty-five extracted first and second human mandibular molars with fully formed apices and without endodontic treatment were selected. The inclusion criteria were mesial roots with Vertucci Type 4^20^, 2 independent foramina, and curvatures between 10 and 20 degrees^21^. To obtain a detailed view of the root canals and confirm the inclusion criteria, the specimens were scanned using a micro-computed tomography device (SkyScan 1174; Bruker microCT, Kontich, Belgium). The scanning parameters were 50 kV and 800 mA, with an isotropic resolution of 19.7 µm, 360° rotation around the vertical axis, and a rotation step of 0.7°. Images were reconstructed using NRecon v.1.6.9 software (Bruker microCT). This process allowed the samples to be grouped into three pairs of teeth based on similar root canal morphology.

### Root canal preparation and filling

A single experienced operator prepared all specimens. Standard access cavities were performed using high-speed diamond burs. Using a dental operative microscope set at 8x magnification, the working length was determined by placing the a size 10 K-file (Dentsply Maillefer, Ballaigues, Switzerland) into the canal so that the tip could be seen through the foramen and then subtracting 0.5 mm.

The root canals were prepared by using NiTi instruments up to size 25.06 using VDW Silver electric motor (VDW, Munich, Germany). The canals were irrigated with 1 mL 1% sodium hypochlorite, and a final rinse was performed with 5 mL 17% EDTA for 3 minutes and 5mL of saline solution. For root canal filling, the single-cone technique was used. A 25.06 gutta-percha cone with an adequate tug-back was selected and coated with zinc oxide and eugenol-based sealer (Endofill, Dentsply, Petrópolis, RJ, Brazil) mixed with Rhodamine B (Sigma-Aldrich, St Louis, USA) at a 1:100 ratio by weight for fluorescence. Coronal accesses were sealed with temporary filling material (Coltosol, Coltene-Whaledent, Cuyahoga Falls, USA), and the teeth were stored at 37ºC and 100% humidity for 7 days to allow complete setting of the sealer.

To obtain the volume of the filling material, the samples were scanned using the micro-CT system (SkyScan 1174v2; Bruker-microCT, Kontich, Belgium) with 50 kV, 800 mA, 0.7º step size rotation, and 19.7 mm voxel resolution. The data were elaborated by the reconstruction software (NRecon v.1.6.3; Bruker-microCT) and the CTan 1.14.4.1 software (Bruker-microCT).

### Retreatment

After evaluation of the initial filing volume, the samples were divided into 3 retreatment groups (Reciproc, Hyflex CM, and ProDesign Duo Hybrid), an n of 15. An aliquot of 0.5 mL xylene solvent (Merck KGaA, Darmstadt, Germany) was placed into the pulp chamber for 30 seconds to soften the gutta-percha at the cervical level before beginning the removal of the root filling.

In the Reciproc group (n = 15), root fillings were removed using R25 Reciproc files (size 25 with a taper of 0.08) (VDW, Munich, Germany) with the respective Reciproc program of the VDW Silver electric motor up to the working length followed by apical enlargement with a R40 file (size 40 with a taper of 0.06).

In the Hyflex CM group (n = 15), root fillings were removed using a size 25 file with a taper of 0.06 with a VDW Silver electric motor in rotary mode up to the working length followed by apical enlargement with a size 40 file with a taper of 0.04, using 500 rpm and 2 N/cm with minimal apical pressure.

For the ProDesign Duo Hybrid group (n = 15), root fillings were removed using a size 25 file with a taper of 0.06 with an Easy Endo SI^®^ motor, in rotary mode (600 rpm and a torque of 2 N/cm) in the cervical portion. The file that reached the working length was used in a reciprocating motion and then a rotary movement was performed again with the 25/.06 at the working length to complete the filling removal. Apical enlargement was then made with a size 40 file with a taper of 0.05.

In all groups, irrigation was performed with 2 mL 1% NaOCl after the use each instrument. NiTi instruments were discarded after four uses or if a visible deformation occurred. Time to reach working length (T1) as well as time needed for complete gutta-percha removal and preparation to size 40 (T2) were recorded. Time for instrument changes and irrigation was not recorded. Total working time was calculated by adding T1 and T2. Removal was considered complete when no residual filling material was observed in the instrument flutes or in the irrigation solution.

The root canals were refilled by the single-cone technique and AH Plus sealer (Dentsply Maillefer, Ballaigues, Switzerland) with fluorescein dye (Sigma-Aldrich, St Louis, USA). Fluorescein was mixed with the sealer at a 1:100 ratio by weight. Coronal accesses were sealed with temporary filling and the teeth were stored at 37ºC and 100% humidity for 7 days to allow complete setting of the sealer.

### Micro-CT scanning procedures

The teeth were scanned 2 times: after the root canal filling and after retreatment. The teeth were placed in the molds and scanned using the same parameters as the initial scan. The images of before and after filling removal were geometrically aligned using the 3D registration option of the DataViewer software v.1.5.1 (Bruker microCT). The scanning and reconstruction parameters used after the root canal filling were the same for all specimens.

The mesiodistal root canal transportation was measured manually in each cross-section evaluated. The formula (M1-M2) - (D1-D2) was used, in which M1 is the mesial edge before filling removal, M2 is the mesial edge after filling removal, D1 is the distal edge before filling removal, and D2 is the distal edge after filling removal. The root canal transportation after retreatment was evaluated in 5 slices: 2, 4, 6, 8, and 10 mm

The volume (mm^3^) of the filling material was measured using the CTAn V.1.14.4 software (Bruker microCT) in three segments from the apical foramen: apical (1–3 mm), middle (3–5 mm), and coronal (5–7 mm). A binary value of the filling material was defined for each segment and this value was applied to the respective segment of the same sample after filling removal.

The root apex was used as the landmark to determine the root canal areas analyzed. The percentage of residual filling material after retreatment was expressed as a percentage of the initial root filling volume.

### Confocal microscopy analysis

After the micro-CT analysis, the specimens were horizontally sectioned at 1 mm from the apex (segment discarded) and then in to slices of 2 mm thickness with a 0.3 mm Isomet saw in a cutting machine (Isomet, Buehler, Lake Bluff, Illinois, USA) under water cooling. The slices of each root canal were evaluated using a confocal inverted microscope Leica TCS-SPE (Leica Microsystems GmbH, Mannheim, Germany) and a method of epifluorescence with wavelengths of absorption and emission to rhodamine B of 540/590 nm and to fluorescein of 536/617 nm. The samples were analyzed at 10 μm below the surface sample with 10x magnification.

To evaluate the volume of filling material that was not removed during retreatment, the LAS x 3D and 2D analysis V1.46r (Leica Microsystems GmbH, Mannheim, Germany) software was used. The scale offered by CLSM images (100 μm) was set in the program and used to measure the total amount of the old material. Once these measurements were obtained, the percentages were calculated for all sections. The volumes were recorded, and the percentage of remnant filling material after each procedure was expressed as a percentage of the initial root-filling material volume.

### Statistical analysis

GraphPad Prism 7 (La Jolla, USA) was used as the analytical tool. Preliminary analysis of data normality was performed with the Shapiro-Wilk test.

The intra-group data regarding the presence or absence of root canal deviation after removal of root canal filling material and re-instrumentation were compared with the parametric paired t-test, since data had a normal distribution.

The working time with NiTi alloys with different heat treatments used in this study was analyzed through parametric ANOVA and Tukey statistical test.

The volume of remaining material in the root canals was compared with nonparametric Kruskal-Wallis and Dunn tests. For the intra-group comparison between different slices (2, 4 and 6 mm), the data were submitted to nonparametric Friedman and Dunn tests. The significance level of 5% (p < 0.05) was considered.

## Results

During the retreatment procedure, two instruments of the Hyflex CM group fractured during the third use and the specimens were discarded.

**Figure 1:**
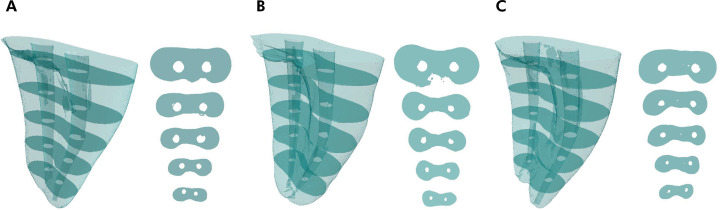
Representative slices at 2, 4, 6, 8, and 10 mm from the apex in Reciproc group (A), Predesign Duo Hybrid (B), and Hyflex (C).

Canal transportation was low in all groups, being directed towards the furcation region in segments 4, 6, 8, and 10 and in the opposite direction in the 2-mm segment (Figure 1). However, there was no significant difference between segments and between systems for each segment (Table 1).

**Table 1:** Mean, minimum and maximum canal transportation (mm) in different slices after endodontic retreatment.

Variable	Canal transportation (mm)				
	2 mm	4 mm	6 mm	8 mm	10 mm
Reciproc	-0.01 (-0.04 / 0.02)^a, A^	0.01 (-0.01 / 0.03)^a, A^	0.02 (-0.02 / 0.08)^a, A^	0.03 (-0.01 / 0.07)^a, A^	0.03 (-0.02 / 0.06)^a, A^
Hyflex	-0.01 (-0.01 / 0.04)^a, A^	0.01 (-0.01 / 0.05)^a, A^	0.02 (-0.02 / 0.06)^a, A^	0.04 (-0.02 / 0.06)^a, A^	0.02 (-0.01 / 0.07)^a, A^
ProDesign Duo Híbrido	-0.03 (-0.02 / 0.05)^a, A^	-0.01 (-0.01 / 003) ^a, A^	0.03 (-0.01 / 0.05) ^a, A^	0.04 (-0.01 / 0.04)^a, A^	0.03 (-0.02 / 0.05)^a, A^

Same superscript lowercase letters in each column indicate no statistically significant difference in the different sections (p > 0.05). Same superscript uppercase letters in each row indicate no statistically significant difference between the groups (p > 0.05).

The mean time to perform endodontic retreatment with the different instruments is shown in Table 2. The time required to remove the filling material (T1) from the root canals was significantly higher in the Hyflex CM group. The Reciproc group presented significantly less time for apical enlargement (T2) and total time (T1 + T2) for root canals retreatment (p < 0.05).

**Table 2:** Mean (± standard deviation – SD) time required for removal of the endodontic filling material (T1) and reinstrumentation (T2), and total treatment time (T1 + T2).

Variable	Retreatment time		
	T1 (s)	T2 (s)	TOTAL (T1 + T2)
Reciproc	70.46 ±13.11^a^	22.32 ± 4.60^b^	92.78 ± 15.58^c^
Hyflex	117.9 ± 27.01^b^	32.98 ± 14.69^a^	150.9 ± 26.96^b^
ProDesign Duo Híbrido	86.98 ±17.8^a^	34.42 ± 4.56^a^	121.4 ± 16.75^a^

Different superscript lowercase letters in each column indicate statistically significant difference (p < 0.05).

**Figure 2:**
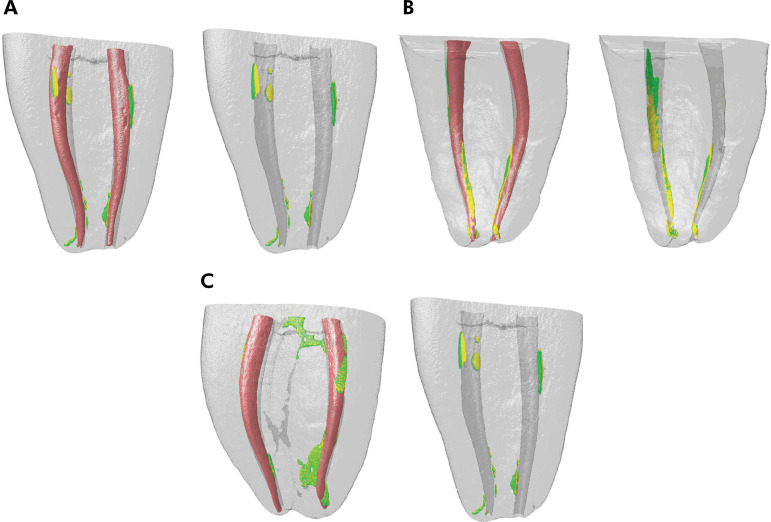
Micro-CT reconstructions of overlay images of the obturation (red) and retreatment with 0.25 instruments (yellow) and with 0.40 instruments (green) in Reciproc group (A), Predesign Duo Hybrid (B), and Hyflex (C).

**Figure 3:**
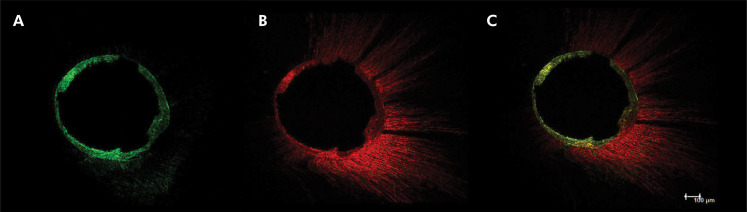
CLSM illustration: a. presence of Endofill in dentinal tubules (red) in the entire circumference of the root canal and gutta-percha cones (black color); b. discrete quantity of AH Plus (green); c. overlay of the images.

Remaining filling material was observed in all specimens (Figure 2). Mean percentages of residual material are shown in Table 3 in different levels. All retreatment techniques left 5.56–8.97% (confocal analysis) (Figure 3) and 5.92–8.52% (micro-CT analysis) of the filling material in the root canal. The ProDesign Duo Hybrid group had a lower percentage of filling material in all levels compared to the other groups, but with no statistical significance (p > 0.05).

**Table 3:** Mean volume (mm3) and standard deviation (SD) of remaining filling material in different root canal slices.

Variable	Volume (%)					
	CLSM analysis			Micro-CT analysis		
	1–3 mm	3–5 mm	5–7 mm	1–3 mm	3–5 mm	5–7 mm
Reciproc	8.21 ± 12.06^a, A^	7.68 ± 6.02^a, A^	7.33 ± 5.15^a, A^	8.03 ± 5.12^a, A^	7.99 ± 5.10^a, A^	6.47 ± 4.80^a, A^
Hyflex	8.97 ± 6.73^a, A^	8.87 ± 6.00^a, A^	7.38 ± 5.89^a, A^	8.19 ± 5.47^a, A^	8.52 ± 5.89^a, A^	8.11 ± 5.25^a, A^
ProDesign Duo Híbrido	6.37 ± 4.11^a, A^	5.74 ± 4.29^a, A^	5.56 ± 3.97^a, A^	7.86 ± 5.20^a, A^	7.00 ± 6.70^a, A^	5.91 ± 4.43^a, A^

Same superscript lowercase letters in each column indicate no statistically significant difference in the different sections (p > 0.05). Same superscript uppercase letters in each row indicate no statistically significant difference between the groups (p > 0.05).

## Discussion

The aim of endodontic retreatment is to completely remove the previous filling material, but no system is capable of achieving this,^6-12,18-20^ which was also evidenced in this study. In addition, the anatomy of the root canal can make this removal even more difficult.

In this study, similar first mandibular molars with mesial roots with two canals that presented distinct foramens and without isthmuses were selected. This type of root canal configuration provided both a similar mesial and distal wall thicknesses and canal volumes. However, in curved root canals, some errors might occur during the process, including canal transportation, ledge formation, and/or canal perforation^23-25^.

The use of NiTi files reduce the time and enhance the safety of the retreatment procedure, so that rotary and reciprocating instruments have been widely used to preserve the original shape of the root canal as much as possible.^9,10,25-27^ In this study, instruments with different kinematics and different heat treatments were used, including rotary files, reciprocating instruments, and a combination between the two motions.

This study showed no significant differences in canal transportation between the groups at the different levels. This might be attributed to the structural properties of these files. The S-shaped cross-section with two cutting edges almost perpendicular in the Reciproc file and the thermomechanical treatment in the Hyflex CM and ProDesign Duo Hybrid files result in an increase in flexibility and centering ability.^28,29^ In all groups, the 4, 6, 8, and 10 mm sections were transported to the critical zone (towards the furcation), while the section 2 mm from apex was transported in the opposite direction to the furcation. The transportation of the canal did not seem to affect the results, as a similar volume of remaining filling material was found in the groups. Several factors influence the level and direction of canal transportation in addition to the metallurgic properties^17,30^and the cross-section of the file.^31^ These include operator technique, canal system anatomy, canal curvature, and dimension of the file^31^. Moreover, in the single cone obturation technique, the gutta-percha cone itself serves as a guide for the instrument along the root canal, which facilitates its removal and minimizes root canal transportation. When using thermoplastic filling techniques, which provides better filling of the root canal, a greater amount of the material remains in the canal after retreatment compared to cold filling techniques.^32-34^

The file metal alloy and cross-section had no influence on the results, as the cutting was similar between the groups. Although the systems have different tapers, the dilation in the cervical and middle thirds was small compared to before filling, and the small increase was not enough to cause significant wear. The results revealed that Reciproc was faster in removing endodontic filling than rotary files, as also shown in others studies.^35,36^ The Hyflex group was slower than the others, even when using a “single file”, probably due to the thermomechanical treatment it undergoes, making it more flexible and reducing its cutting ability^37^.

Although the instruments were efficient in removing a large amount of gutta-percha, none of the techniques was able to completely remove the filling material from the root canal walls, in agreement with previous studies.^9,10,12,27,38^ The presence of filling remnants in all groups could be attributed to the oval shape of the canals, in which the flatter regions are protected from the action of the file.^26,33,39^

Under the experimental conditions, ﬁlling material removal was efﬁcient in all evaluated segments in the 3 groups. Alloy type and the kinematics of the instruments did not influence the removal of the filling material, corroborating previous studies.^27,40^ However, another study found better results with the adaptive motion than the rotational movement using the same instrument.^19^ The green dye showed areas of the dentinal tubules filled with the AH Plus sealer, suggesting that there was adequate cleaning of the region, corroborating previous studies,^41^ but there was less penetration compared to the initial obturation.

The satisfactory performance of ProDesign Duo Hybrid was probably due to the association of two different movements (rotational and reciprocating) during endodontic retreatment, which keeps the file in motion longer, enhancing the removal of the filling material from the canal.

## Conclusion

Reciproc, Hyflex CM, and ProDesign Duo Hybrid systems were similar in the canal transport parameter and were considered safe for the retreatment of curved root canals. Canal preparation with the Reciproc system was faster than with the other systems. None of the retreatment techniques completely removed the canal fillings. The combination of reciprocating and rotary files in the retreatment of curved canals was as efficient as rotary and reciprocating motion alone.
